# Supplementary material for: Isoform cell-type specificity in the mouse primary motor cortex
Source: Nature. 2021 Oct 6;598(7879):195–9. doi: 10.1038/s41586-021-03969-3 (PMC8494650; doi:10.1038/s41586-021-03969-3)
Supplement: Supplementary file 1 — This file contains Supplementary Figs. 1–7. [file 41586_2021_3969_MOESM1_ESM.pdf]

nature portfolio

https://doi.org/10.1038/s41586-021-03969-3

Supplementary information

Isoform cell-type specificity in the mouse primary motor cortex

In the format provided by the authors and unedited

Supplementary Figures

Isoform cell type specificity in the mouse primary motor cortex

A. Sina Booeshaghi[1], Zizhen Yao[2], Cindy van Velthoven[2], Kimberly Smith[2], Bosiljka Tasic[2], Hongkui Zeng[2], and Lior Pachter[3,4]

1. Department of Mechanical Engineering, California Institute of Technology, Pasadena, California
2. Allen Institute for Brain Science, Seattle, Washington
3. Division of Biology and Biological Engineering, California Institute of Technology, Pasadena, California
4. Department of Computing and Mathematical Sciences, California Institute of Technology, Pasadena, California

Address correspondence to lpachter@caltech.edu

Note: The captions of the Supplementary Figures contain links to the code used to make the figures.

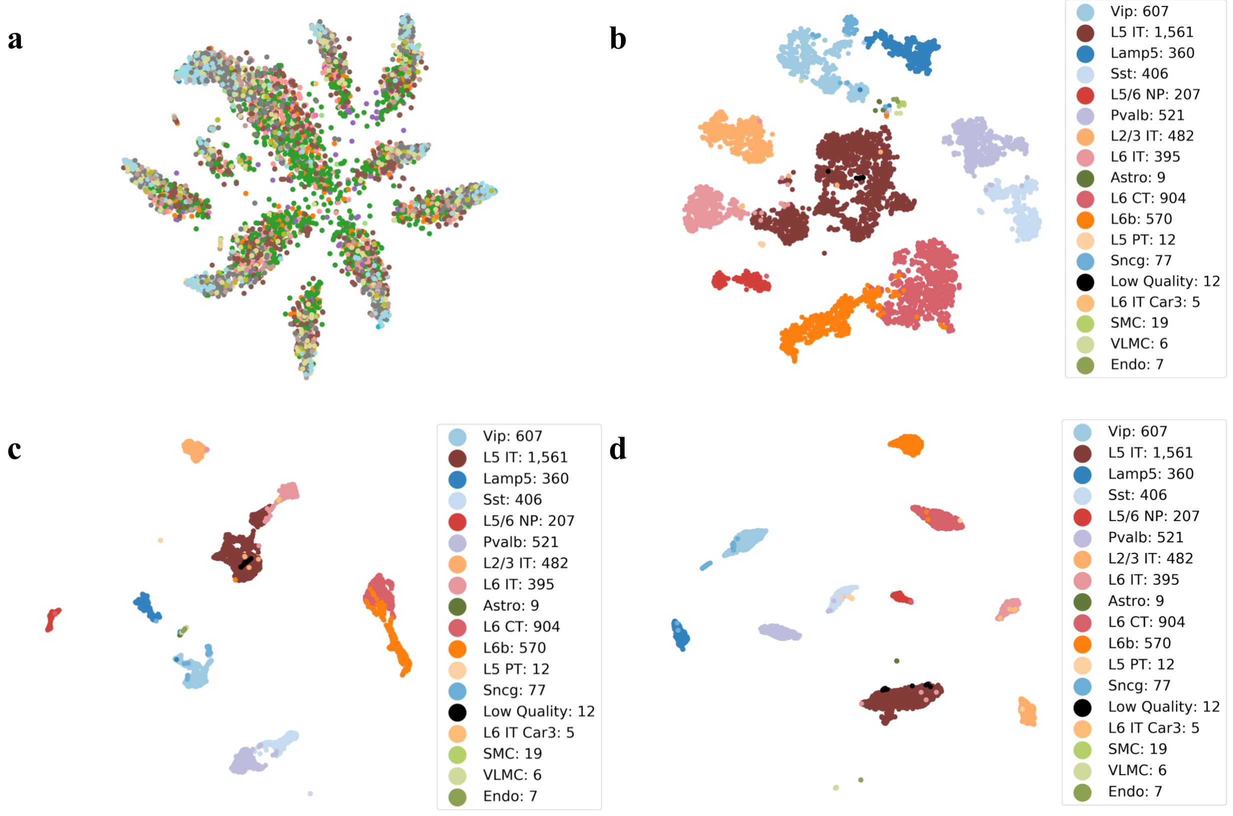

Supplementary Figure 1: a) NCA on the scaled log1p normalized SMART-Seq gene matrix with the subclass labels permuted randomly to ten components, followed by t-SNE to dimension two. The lack of separation of cells by label demonstrates that the NCA procedure is not overfitting the data. b) Truncated singular value decomposition (TSVD) on the scaled log1p normalized SMART-Seq gene matrix to 50 components followed by t-SNE to two dimensions. The numbers next to each subclass label indicate the number of cells in the subclass. c) TSVD on the scaled log1p normalized SMART-Seq gene matrix to 50 components followed by uniform manifold approximation and projection (UMAP) to two dimensions. The numbers next to each subclass label indicate the number of cells in the subclass. d) Ten components of the NCA on the scaled log1p normalized SMART-Seq gene matrix followed by UMAP to two dimensions. The numbers next to each subclass label indicate the number of cells in the subclass. [Code a, Code b, Code c, Code d]

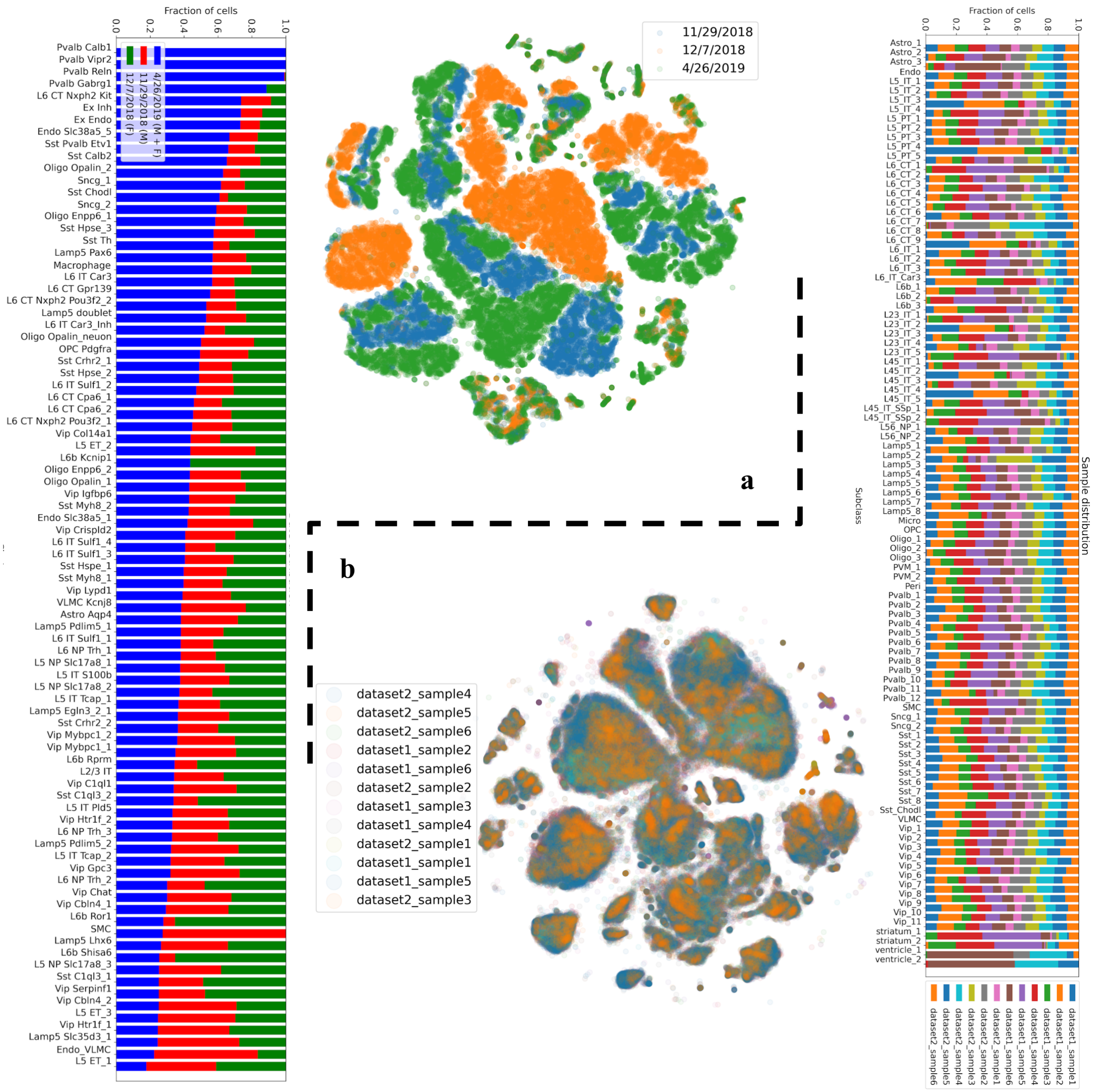

Supplementary Figure 2: a) Fraction of cells per cluster in the 10xv3 dataset originating from a specific processing date. 24,348 male cells were assayed on 11/29/2018, 32,145 female cells were assayed on 12/7/2018, and 18,140 male cells and 15,398 female cells were assayed on 4/26/2019. The accompanying TSVD to 50 components followed by t-SNE to two dimensions of the 10xv3 data shows each cell painted by the date it was assayed. The separation by date indicates a strong batch effect by date. b) t-SNE to two components of the MERFISH dataset where each cell is painted by the sample it originated from,

alongside the fraction of cells per cluster in the MERFISH dataset originating from a specific sample.

[Code a, Code b]

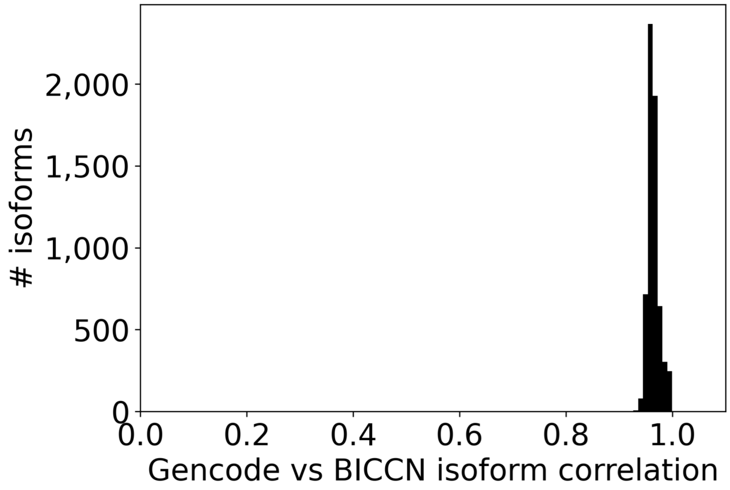

Supplementary Figure 3: Comparison of isoform quantifications obtained with respect to the Gencode M25 and BICCN annotations. The average Pearson correlation across 107,639 isoforms was 0.965. Code

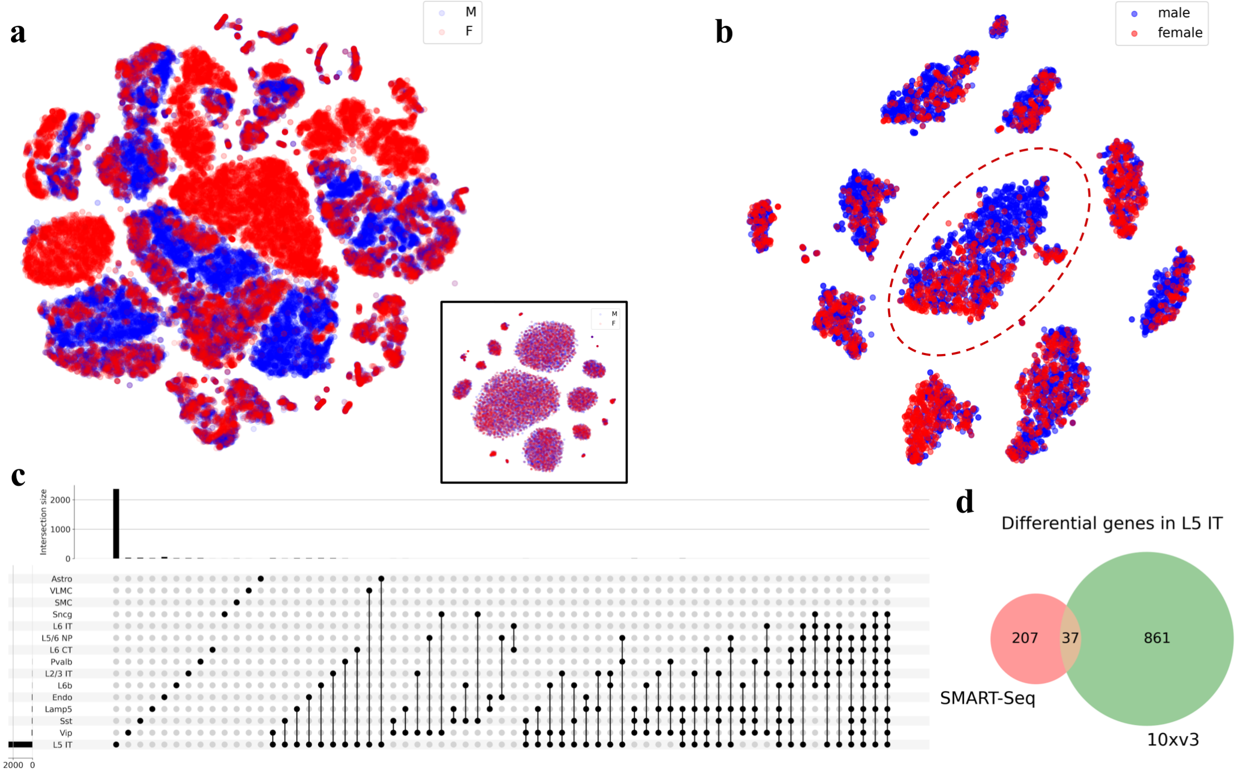

Supplementary Figure 4: a) TSVD followed by t-SNE of all of the 10xv3 data. The clusters segregate by sex. The inset graph shows NCA followed by t-SNE on a subset of the 10xv3 data. The subset graph is showing only the cells assayed on 4/26/2019. The clusters do not appear to segregate by sex. b) NCA followed by t-SNE of the SMART-Seq dataset. Each cell is painted by the sex of the animal it originated from. The L5 IT subclass is enclosed with a dotted red ellipse. c) Upset plot showing the number of isoforms that are unique and shared between subclasses after differential expression based on sex within each subclass for the SMART-Seq dataset. d) Venn diagram of the number of differential genes shared between the SMART-Seq and 10xv3 quantifications for the L5 IT subclass. [Code a (subset), Code b, Code c, Code d]

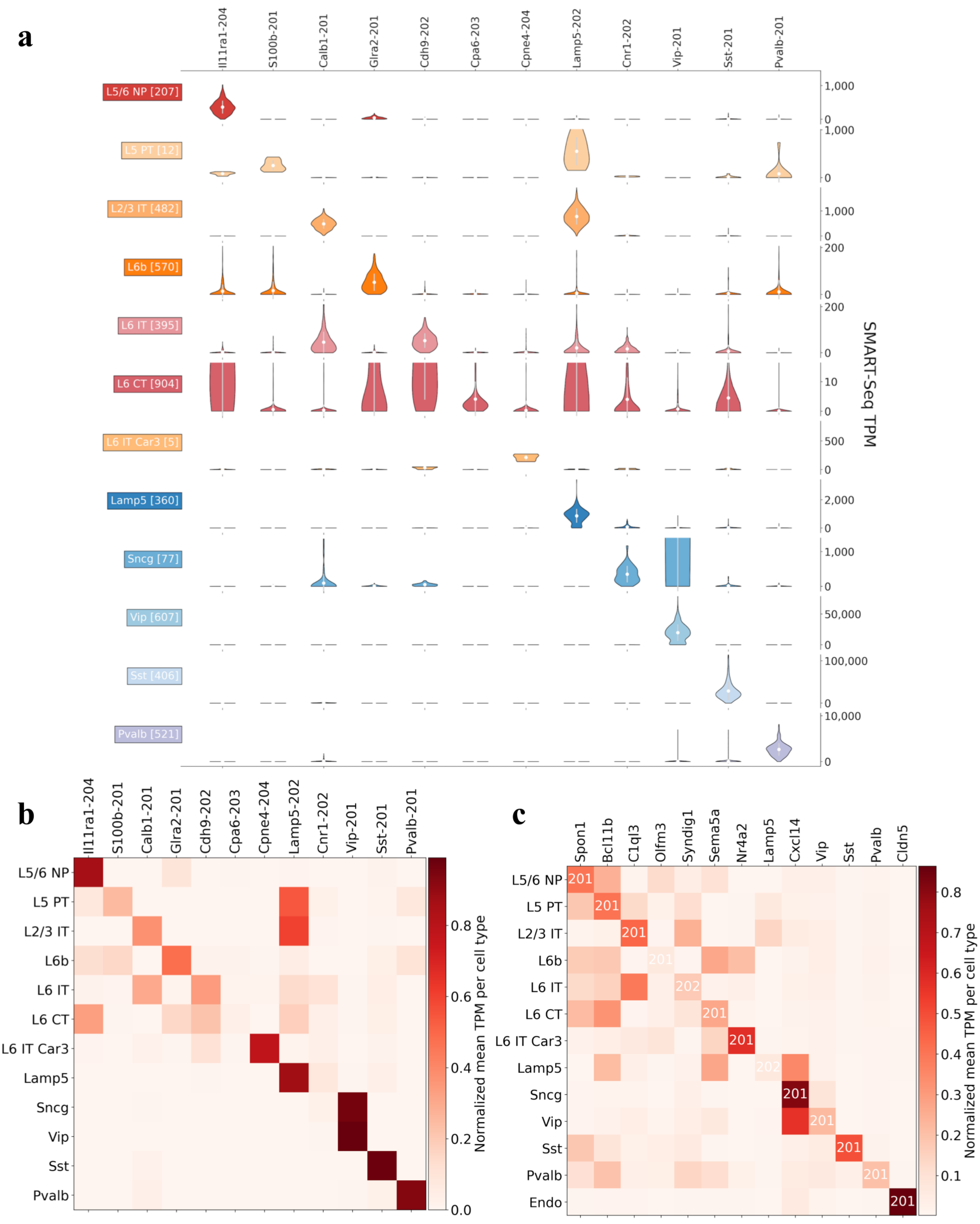

Supplementary Figure 5: a) A sample from an isoform atlas displaying isoform markers differential with respect to subclasses. Each row corresponds to one subclass, and each column corresponds to one

isoform. SMART-Seq isoform abundance estimates are in TPM units, and each column is scaled so that the maximum TPM is 4 times the mean of the isoform specific for that row's cluster. The white circles within the violin plots represent the mean and the white bars represent + / - one standard deviation. b) A sample from an isoform atlas displaying isoform markers differential with respect to subclasses. Each row corresponds to one subclass, and each column corresponds to one isoform. SMART-Seq isoform abundance estimates are in TPM units, and each row is scaled by the sum of the mean expressions of each isoform for all cells in that subclass. c) A sample from a spatial-isoform atlas inferring spatial isoform markers for cell types with known spatial locations and known gene markers. Each row corresponds to one subclass, and each column corresponds to one gene with the inferred differential isoform from that gene, determined by the SMART-Seq data, along the diagonal. SMART-Seq isoform abundance estimates are in TPM units, and each row is scaled by the sum of the mean expressions of each gene for all cells in that subclass. [Code a, Code b, Code c]

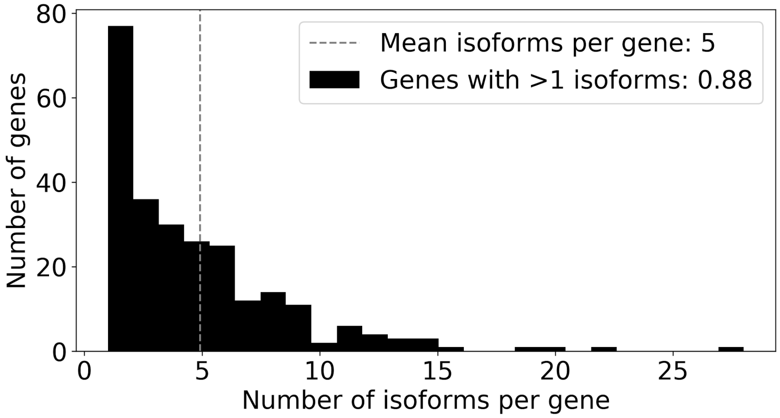

Supplementary Figure 6: The distribution of the number of isoforms per gene for all of the genes in the MERFISH dataset. 88% of genes in the MERFISH dataset have more than one isoform. Code

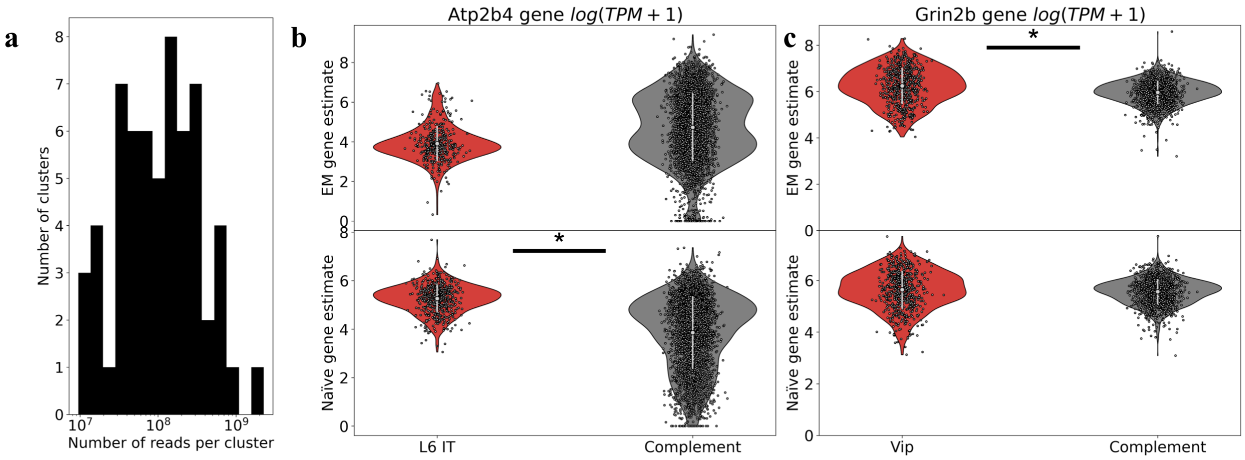

Supplementary Figure 7: a) Distribution of read depth per SMART-seq cell showing sufficient depth for accurate quantification with the expectation-maximization (EM) algorithm. b) Comparison of two quantifications approaches: EM gene quantification where isoform abundances are estimated, and then added up to obtain a gene abundance estimate, versus naive quantification in which read counts are collated by gene locus (* indicates statistically significant difference p<0.01). In this case naïve quantification introduces a possibly incorrect gene marker for the Pvalb subclass. c) In this case naïve quantification does not identify a gene marker for the L6 CT subclass. The white circles within the violin plots represent the mean and the white bars represent + / - one standard deviation. [Code a, Code b,c]
