# Supplementary material for: Isoform cell-type specificity in the mouse primary motor cortex
Source: Nature. 2021 Oct 6;598(7879):195–9. doi: 10.1038/s41586-021-03969-3 (PMC8494650; doi:10.1038/s41586-021-03969-3)
Supplement: Supplementary file 2 — Reporting Summary [file 41586_2021_3969_MOESM2_ESM.pdf]

nature research

Corresponding author(s): Lior Pachter
Last updated by author(s): Jul 21, 2021

Reporting Summary

Nature Research wishes to improve the reproducibility of the work that we publish. This form provides structure for consistency and transparency in reporting. For further information on Nature Research policies, see our Editorial Policies and the Editorial Policy Checklist.

Statistics

For all statistical analyses, confirm that the following items are present in the figure legend, table legend, main text, or Methods section.

n/a	Confirmed	
☐	☒	The exact sample size (n) for each experimental group/condition, given as a discrete number and unit of measurement
☐	☒	A statement on whether measurements were taken from distinct samples or whether the same sample was measured repeatedly
☐	☒	The statistical test(s) used AND whether they are one- or two-sided<br>*Only common tests should be described solely by name; describe more complex techniques in the Methods section.*
☐	☒	A description of all covariates tested
☐	☒	A description of any assumptions or corrections, such as tests of normality and adjustment for multiple comparisons
☐	☒	A full description of the statistical parameters including central tendency (e.g. means) or other basic estimates (e.g. regression coefficient) AND variation (e.g. standard deviation) or associated estimates of uncertainty (e.g. confidence intervals)
☐	☒	For null hypothesis testing, the test statistic (e.g. F, t, r) with confidence intervals, effect sizes, degrees of freedom and P value noted<br>*Give P values as exact values whenever suitable.*
☒	☐	For Bayesian analysis, information on the choice of priors and Markov chain Monte Carlo settings
☒	☐	For hierarchical and complex designs, identification of the appropriate level for tests and full reporting of outcomes
☐	☒	Estimates of effect sizes (e.g. Cohen's d, Pearson's r), indicating how they were calculated

Our web collection on statistics for biologists contains articles on many of the points above.

Software and code

Policy information about availability of computer code

Data collection	No software was used for data collection.
Data analysis	Anndata 0.7.1<br>bustools 0.39.4<br>awk (GNU awk) 4.1.4<br>grep (GNU grep) 3.1<br>kallisto 0.46.1<br>kb_python 0.24.4<br>Matplotlib 3.0.3<br>Numpy 1.18.1<br>Pandas 0.25.3<br>Scanpy 1.4.5.post3<br>Scipy 1.4.1<br>sed (GNU sed) 4.4<br>sklearn 0.22.1<br>statsmodels 0.12.1<br>tar (GNU tar) 1.29<br>umap 0.3.10

For manuscripts utilizing custom algorithms or software that are central to the research but not yet described in published literature, software must be made available to editors and reviewers. We strongly encourage code deposition in a community repository (e.g. GitHub). See the Nature Research guidelines for submitting code & software for further information.

Data

Policy information about availability of data

All manuscripts must include a data availability statement. This statement should provide the following information, where applicable:

- Accession codes, unique identifiers, or web links for publicly available datasets
- A list of figures that have associated raw data
- A description of any restrictions on data availability

The single-cell RNA-seq data used in this study was generated as part of the BICCN consortium20. The 10xv3 and SMART-Seq data can be downloaded from http://data.nemoarchive.org/biccn/lab/zeng/transcriptome/scell/. The MERFISH data is available at https://caltech.box.com/shared/static/dzqt6ryytmjbgyai356s1z0phtnsbaol.gz. All cell annotations and cluster labels are available at https://github.com/pachterlab/BYVSTZP_2020/tree/master/reference.

Field-specific reporting

Please select the one below that is the best fit for your research. If you are not sure, read the appropriate sections before making your selection.

☒ Life sciences ☐ Behavioural & social sciences ☐ Ecological, evolutionary & environmental sciences

For a reference copy of the document with all sections, see nature.com/documents/nr-reporting-summary-flat.pdf

Life sciences study design

All studies must disclose on these points even when the disclosure is negative.

Sample size	No explicit calculations were performed to determine sample size. We analyzed 6,160 mouse primary motor cortex cells assayed with SMART-Seq, 280,327 cells assayed with MERFISH, and 94,162 cells assayed with 10x Genomics Chromium v3. We analyzed both male and female mice to understand differences in gene and isoform expression. The sample size for differential expression was set to be such that 90% of cells in a cluster have a non-zero expression of the tested gene. The smallest cluster size contained 7 cells with all cells having non-zero expression of the tested genes. We computed error bars for all tests to ensure that sample sizes were sufficient.
Data exclusions	We excluded a subset of the 10xv3 data that was collected on separate dates due to a batch effect. We describe our rationale for exclusion in detail in the manuscript. In short, specific cell-types were enriched for within those batches.
Replication	We validated each technology by comparing average cell type expression between MERFISH, SMART-Seq, and 10xv3 at the gene level and at the isoform level for SMART-Seq and 10xv3. These comparisons are replicated and consistent across the three heirarchies: class, subclass, and cluster. We describe the methods in the manuscript.
Randomization	All cells that passed quality control were analyzed equally with no sub-sampling therefore there was no requirement for randomization.
Blinding	Cell type assignment was not blinded due to practical constraints.

Reporting for specific materials, systems and methods

We require information from authors about some types of materials, experimental systems and methods used in many studies. Here, indicate whether each material, system or method listed is relevant to your study. If you are not sure if a list item applies to your research, read the appropriate section before selecting a response.

Materials & experimental systems

n/a	Involved in the study	
☒	☐	Antibodies
☒	☐	Eukaryotic cell lines
☒	☐	Palaeontology and archaeology
☐	☒	Animals and other organisms
☒	☐	Human research participants
☒	☐	Clinical data
☒	☐	Dual use research of concern

Methods

n/a	Involved in the study	
☒	☐	ChIP-seq
☒	☐	Flow cytometry
☒	☐	MRI-based neuroimaging

Animals and other organisms

Policy information about studies involving animals; ARRIVE guidelines recommended for reporting animal research

Laboratory animals	Mice were provided food and water ad libitum and were maintained on a regular 12-h day/night cycle at no more than five adult animals per cage. For this study, we enriched for neurons by using Snap25-IRES2-Cre mice58 (MGI:J:220523) crossed to Ai1459 (MGI:

	J:220523), which were maintained on the C57BL/6J background (RRID:IMSR_JAX:000664). Animals were euthanized at 53–59 days of postnatal age. Tissue was collected from both males and females (scRNA SMART, scRNA 10x v3). (MERFISH, companion paper) Adult C57BL/6 male mice aged 57-63 days were used in this study. Animals were maintained on a 12 hour:12 hour light/dark cycle (2pm-2am dark period) with ad libitum access to food and water.
Wild animals	No wild animals were used in this study.
Field-collected samples	No field-collected samples were used in this study.
Ethics oversight	All procedures were carried out in accordance with Institutional Animal Care and Use Committee protocols at the Allen Institute for Brain Science.

Note that full information on the approval of the study protocol must also be provided in the manuscript.
